# Computational analysis of MHD ternary hybrid nanofluid flow with thermochemical reactions through a porous medium on a rotating stretching sheet

**DOI:** 10.1186/s11671-025-04353-0

**Published:** 2025-09-25

**Authors:** A. B. Sadiya, G. Sucharitha

**Affiliations:** Department of Mathematics, School of Advanced Sciences, Vellore Institute of Technology, Vellore, Tamil Nadu 632014 India

**Keywords:** Ternary hybrid nanofluid, Darcy–Forchheimer, Thermal radiation, Endothermic/exothermic chemical reaction, Rotating stretching sheet

## Abstract

Inefficient thermal transmission in heat exchangers requires creative solutions. Ternary hybrid nanofluids have evolved to offer improved thermal efficiency compared to standard nanofluids. The current study involves a ternary hybrid nanofluid of copper oxide (CuO), titanium dioxide (TiO_2_), and silver (Ag) nanoparticles suspended in a base fluid of water-ethylene glycol (50–50%) (H_2_O–C_2_H_6_O_2_) to enhance thermal efficiency. This comprehensive analysis aims to provide insights into the heat transfer behaviour of a ternary hybrid nanofluid flow through a porous medium, considering the magnetic field effects in the momentum equation, exothermic/endothermic (Thermochemical) reactions in the energy equation, and activation energy in the concentration equation, respectively, on a rotating stretching sheet. Partial differential equations (PDEs) govern the flow problem. PDEs are converted to Ordinary differential equations (ODEs) using a suitable similarity transformation to aid solution. The linearised equations are solved numerically using MATLAB’s “bvp4c” boundary value problem solver. Variations in the velocity, temperature and concentration profiles due to various parameters are presented graphically. The results show that increasing M and Fr values increases $\theta$ profile by 1.2% and 0.85% respectively. Whereas the overall increase in the heat transfer is 6.65% and mass transfer is 1.86%, making this a substantial contribution to our work. This research will benefit manufacturers of cosmetics, hydraulic fluids, and fibreglass. Furthermore, the findings are supported by the available literature in specific instances, and they exhibit a strong concordance.

## Introduction

One of the most essential attributes of fluids in engineering and industrial applications is their ability to transfer heat. This attribute is widely employed in various processes involved in power generation, plastic processing, semiconductor manufacturing, solar thermal energy, refrigerating systems in power plants, oilfield applications, drug delivery, microelectronics, and chemical processes. Water, ethanol, ethylene glycol, oil, molten salts, air, steam, and refrigerants are some of the media employed in the heat transfer process. A ternary hybrid nanofluid emerged to overcome the challenges faced by traditional fluids. Ternary hybrid nanofluids represent a novel class of heat transfer fluids, comprising a base fluid infused with a combination of three distinct types of nanoparticles, typically metals or metal-oxide particles. Saghir and Bayomy [1] experimented with a ternary mixture for heat enhancement and storage in a circular pipe in 2018. Later, these fluids gained importance in the early 20 s, and researchers started to work in this field due to their wide applications and diverse thermophysical properties. Sahoo and Kumar determined the correlation of viscosity and Thermo-hydraulic characteristics of radiators using ternary hybrid nanofluid [2]. Abbas et al. [3] carried out an analysis of ternary nanofluids using ANN. Followed by a sensitivity analysis and thermo-economic performance of a ternary hybrid nanofluid [4]. Shah et al. used mono and hybrid nanoparticles for the optimization of the heat transfer across a stretching cylinder [5]. The trihybrid nanofluid flow between the conical gap of a rotating disk and a cone using blood as base fluid was also studied [6]. Furthermore, the combination of SWCNT-MWCNT was utilised for analysing the heat transfer dynamics of the nanofluid [7].

Ethylene glycol–water [8] is a commonly used base fluid in heat transfer applications due to its excellent antifreeze properties and relatively high thermal conductivity. They are widely used in solar thermal systems and heat exchangers due to their superior heat transfer capabilities, as well as in electronic cooling devices to lower the temperature and extend the lifespan of electronic devices. Yang et al. utilised water/ethylene glycol-based mono, binary, and ternary hybrid nanoparticles to predict their thermophysical characteristics using artificial neural networks (ANN) [9]. Saleh and Sundar [10] used nanodiamond + Fe_3_O_4_ hybrid nanofluids with ethylene glycol/water-based nanofluid for entropy generation and exergy efficiency. Diverse works using distilled water/ethylene glycol-based nanofluids have been studied for outperforming thermal efficiency using a convergent-divergent nozzle [11], with graphene oxide/MXene nanoparticles [12].

Various experiments have been conducted on a stretching sheet for boundary layer circulation flow due to its extensive applications in industries, including robotics, energy storage in supercapacitors, the manufacturing of shape-memory polymers, biomaterials and tissue engineering, environmental engineering, chemical engineering, and aerospace applications. Wang first examined the characteristics of rotating fluid over a stretching sheet [13, 14]. Later, numerous explorations emerged in this area to enhance the heat and mass transfer rate, leading to significant advancements, including those related to stagnation points, porous media, activation energy, and various substantial effects. Majeed [15] numerically analysed the flow of rotating carbon nanotube fluid over a permeable stretching sheet. The MHD boundary layer analysis of dusty hybrid nanofluids over a porous medium [16], including the effects of shape factor and thermophysical properties on stagnation point flow [17], was investigated. Whereas, insights were gained using the non-Newtonian Powell-Eyring fluid model [18] and the Maxwell fluid model [19], incorporating viscous dissipation and variable thermal conductivity [20], as well as autocatalytic chemical reactions [21]. Furthermore, the different nanoparticle combinations using anisotropic slip [22], Darcy–Forchheimer [23], accompanied by Arrhenius activation energy [24], stagnation point flow of Riga plate [25], with thermal radiation effects [26] were studied.

The research presented in the literature review serves as a source of inspiration for the current study. The previous studies presented by Shah and Awan and Madiwal et al. serve as the primary reference, where this study lacks in the investigation of the integrated effect of Darcy–Forchheimer with stagnation point and radiation effects. Hence, the current study investigates the combined impact of these factors on a ternary nanofluid flow through a porous medium over a rotating stretching sheet. We have also utilised a unique nanoparticle combination, which has significant applications in polymer and plastic film manufacturing, electronic device cooling, petroleum and chemical processing, advanced heat transfer in rotating machinery, heat exchangers, and biomedical and pharmaceutical processing. This extensive research aims to enhance thermal conductivity by investigating the effects of various parameters on concentration, temperature, and velocity profiles, given their physical implications.

## Problem formulation

**Fig. 1:**
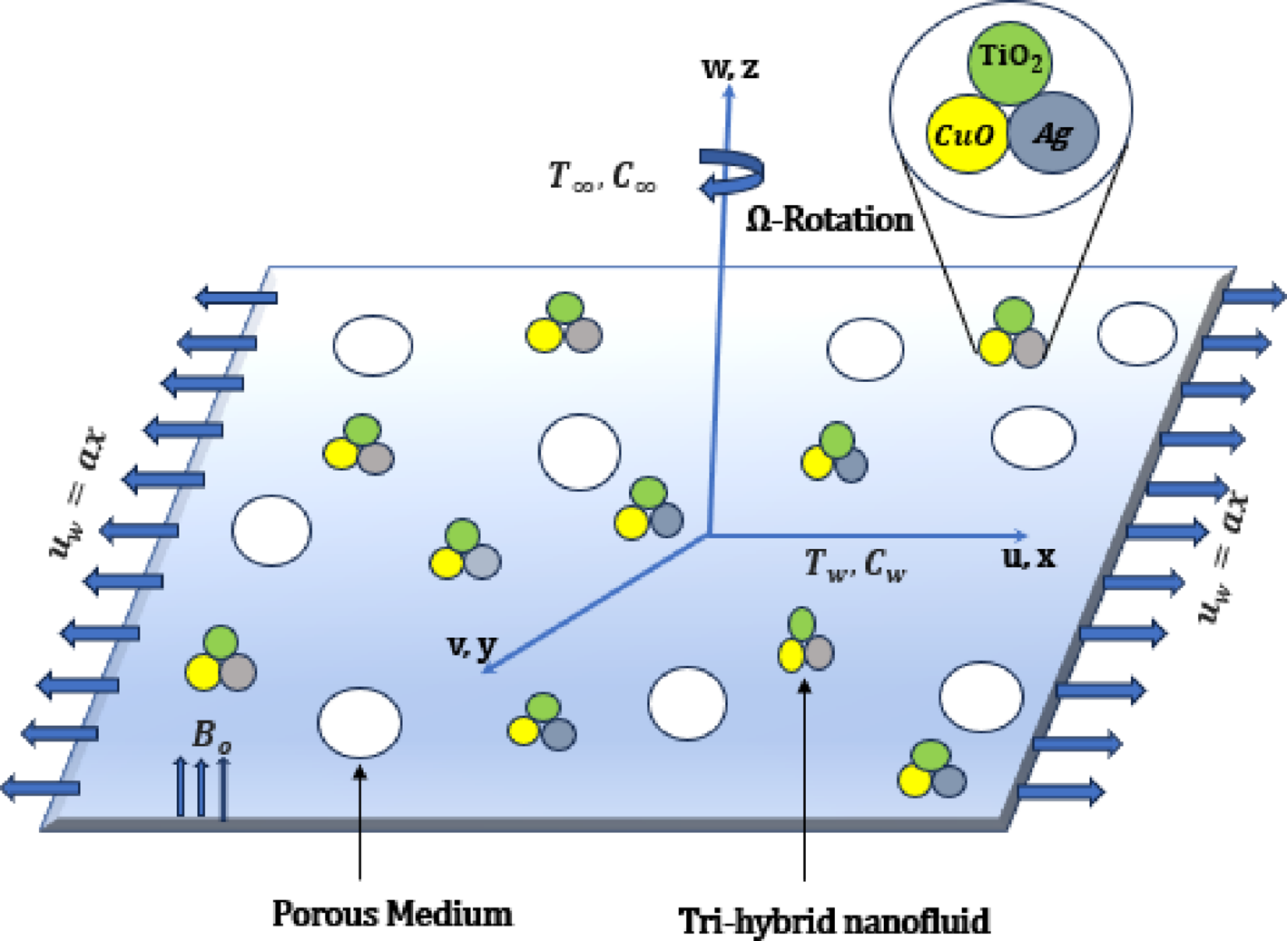
Geometry of the flow problem

A three-dimensional, steady, laminar, and incompressible boundary layer stagnation-point flow is considered, where a ternary hybrid nanofluid flows over a rotating linear stretching sheet embedded with a porous medium. Furthermore, the magnetic field, Darcy–Forchheimer effect in the momentum equation, thermal radiation effects and endothermic-exothermic (Thermochemical) chemical reactions in the energy equation and activation energy in the concentration equation are considered, respectively. Figure 1 illustrates the schematic flow setup for the current flow model. Meanwhile, the problem sketch is bounded by the Cartesian coordinate system *x*, *y*, and *z*, with *u*, *v*, and *w* representing the appropriate velocity components of these flow directions. The flow is confined to $z\ge 0$. Assuming the sheet’s surface expands with uniform stretching velocity, ${u}_{w}=ax$, where $a$ represents the stretching rate factor. The ambient or far-field velocity of the ternary nanofluid is defined as ${u}_{e}=bx$, where $b$ represents the constant stagnation ratio factor. Also, the sheet is rotating with the angular velocity $\Omega ,$ along the *z*-axis. Additionally, the temperature and concentration near the wall are expressed by ${T}_{w}$ and ${C}_{w}$, whereas ${T}_{\infty }$ and ${C}_{\infty }$ represents the ambient temperature and concentration of the nanofluid, respectively.

The governing equations of the flow problem are given below [27, 28]

1 $$ \frac{\partial u}{{\partial x}} + \frac{\partial v}{{\partial y}} + \frac{\partial w}{{\partial z}} = 0, $$

2 $$ \begin{aligned} u\frac{\partial u}{{\partial x}} + & \,v\frac{\partial u}{{\partial y}} + w\frac{\partial u}{{\partial z}} - 2\Omega v = u_{e} \frac{{du_{e} }}{dx} \\ & \, + \vartheta_{THNF} \frac{{\partial^{2} u}}{{\partial z^{2} }} - \frac{1}{{\rho_{THNF} }}\left[ {\frac{{\mu_{THNF} }}{{K_{p} }} + \sigma_{THNF} B_{0}^{2} } \right]\left( {u - u_{e} } \right) - F\left( {u^{2} - u_{e}^{2} } \right), \\ \end{aligned} $$

3 $$ \begin{aligned} u\frac{\partial v}{{\partial x}} + & \,v\frac{\partial v}{{\partial y}} + w\frac{\partial v}{{\partial z}} + 2\Omega u = \vartheta_{THNF} \frac{{\partial^{2} v}}{{\partial z^{2} }} \\ & \, - \frac{1}{{\rho_{THNF} }}\left[ {\frac{{\mu_{THNF} }}{{K_{p} }} + \sigma_{THNF} B_{0}^{2} } \right]v - Fv^{2} , \\ \end{aligned} $$

4 $$ \begin{aligned} u\frac{\partial T}{{\partial x}} + & \,v\frac{\partial T}{{\partial y}} + w\frac{\partial T}{{\partial z}} = \alpha_{THNF} \frac{{\partial^{2} T}}{{\partial z^{2} }} + \frac{{\rho_{F} \varepsilon }}{{\left( {\rho C_{p} } \right)_{THNF} }}Kr^{2} \left( {\frac{T}{{T_{\infty } }}} \right)^{n} exp\left( {\frac{{ - E_{A} }}{KT}} \right)\left( {C - C_{\infty } } \right) \\ & \, + \frac{{16\sigma^{*} }}{{3k^{*} }}\frac{{T_{\infty }^{3} }}{{\left( {\rho C_{p} } \right)_{THNF} }}\frac{{\partial^{2} T}}{{\partial z^{2} }}, \\ \end{aligned} $$

5 $$ u\frac{\partial C}{{\partial x}} + v\frac{\partial C}{{\partial y}} + w\frac{\partial C}{{\partial z}} = D_{F} \frac{{\partial^{2} C}}{{\partial z^{2} }} + Kr^{2} \left( {\frac{T}{{T_{\infty } }}} \right)^{n} exp\left( {\frac{{ - E_{A} }}{KT}} \right)\left( {C - C_{\infty } } \right). $$

with corresponding boundary conditions [27],

6 $$ \left. \begin{gathered} At z = 0: u = ax, v = 0 = w, T = T_{w} , C = C_{w} , \hfill \\ At z \to \infty : u \to bx, v \to 0, T \to T_{w} , C \to C_{w} , \hfill \\ \end{gathered} \right\rangle $$

Utilising the following similarity variables, the governing partial differential equations (PDEs) are transformed into ordinary differential equations (ODEs) [27],

7 $$ \left. \begin{gathered} \eta = z\sqrt {\frac{a}{{\vartheta_{F} }}} , u = axf{\prime} ,\,v = axh, w = - \sqrt {a\vartheta_{F} } f, \hfill \\ \theta = \left( {T - T_{\infty } } \right)\left( {T_{w} - T_{\infty } } \right)^{ - 1} , \chi = \left( {C - C_{\infty } } \right)\left( {C_{w} - C_{\infty } } \right)^{ - 1} . \hfill \\ \end{gathered} \right\} $$

By utilising Eq. (7), Eqns. (2)–(5) can be written as,

8 $$ \begin{aligned} f^{\prime \prime \prime } - \lambda \left( {f^{\prime } - S} \right) + & \,\phi_{1} \phi_{2} \left[ {2Rph + S^{2} + ff^{\prime \prime } - f^{{^{{\prime}{2}} }} + Fr\left( {S^{2} - f^{\prime 2} } \right)} \right] \\ & \, - M\left( {f^{\prime } - S} \right)\phi_{1} \phi_{3} = 0, \\ \end{aligned} $$

9 $$ h^{^{\prime\prime}} - \lambda h - \phi_{1} \phi_{2} \left[ {2Rph{\prime} - fh{\prime} + f{\prime} h + Frh^{2} } \right] - Mh\phi_{1} \phi_{3} = 0, $$

10 $$ \left[ {\frac{{k_{THNF} }}{{k_{F} }} + \frac{4}{3}Rd} \right]\theta^{^{\prime\prime}} + Pr\left[ {Rr\lambda^{*} exp\left( {\frac{ - E}{{\theta \delta + 1}}} \right)\left( {\theta \delta + 1} \right)^{n} \chi } \right] + \theta{\prime} f\phi_{4} = 0, $$

11 $$ \chi^{^{\prime\prime}} + Sc\left[ {f\chi{\prime} - Rrexp\left( {\frac{ - E}{{\theta \delta + 1}}} \right)\left( {\theta \delta + 1} \right)^{n} \chi } \right] = 0. $$

The transformed boundary conditions are,

12 $$ \left. \begin{gathered} \left( {f{\prime} ,\,f,\,h,\,\theta ,\,\chi } \right)_{\eta = 0} = \left( {1,0,0,1,1} \right), \hfill \\ \left( {f{\prime} ,\,h,\,\theta ,\,\chi } \right)_{\eta \to \infty } = \left( {S,0,0,0} \right). \hfill \\ \end{gathered} \right\} $$

Equations (8)–(12) are the non-dimensional equations using the corresponding non-dimensional parameters mentioned in Tables 1 and 2.

**Table 1:** List of non-dimensional parameters and their default values [27–30]

SI. No	Name of the parameter	Expression	Range
1	Porosity parameter	$\lambda = \vartheta_{F} \left( {K_{p} a} \right)^{ - 1} .$	0–1
2	Stretching parameter	$S = ba^{ - 1} .$	0–0.5
3	Rotation parameter	$Rp = \Omega a^{ - 1} .$	0–1
4	Forchheimer number	$Fr = C_{b} \left( {\sqrt {K_{p} } } \right)^{ - 1} .$	0–7
5	Magnetic parameter	$M = \sigma_{F} B_{0}^{2} \left( {\rho_{F} a} \right)^{ - 1} .$	0–4
6	Radiation parameter	$Rd = 4\sigma^{*} T_{\infty }^{3} \left( {k^{*} k_{F} } \right)^{ - 1} .$	0–2
7	Prandtl number	$Pr = \vartheta_{F} \left( {\alpha_{F} } \right)^{ - 1} .$	6.2
8	Reaction rate parameter	$Rr = Kr^{2} a^{ - 1} .$	0–0.5
9	Endothermic/exothermic parameter	$\lambda^{*} = \frac{{\varepsilon \left( {C_{w} - C_{\infty } } \right)}}{{\left( {C_{p} } \right)_{F} \left( {T_{w} - T_{\infty } } \right)}}.$	− 1 ≤ 0 ≤ 1
10	Activation Energy	$E = E_{A} \left( {KT_{\infty } } \right)^{ - 1} .$	0–3
11	Temperature ratio parameter	$\delta = \left( {T_{w} - T_{\infty } } \right)\left( {T_{\infty } } \right)^{ - 1} .$	0–0.5
12	Fitted rate constant	$n.$	1
13	Schmidt number	$Sc = \vartheta_{F} \left( {D_{F} } \right)^{ - 1} .$	0–0.8

**Table 2:** Thermal characteristics of Ternary Hybrid Nanofluids [30]

SI. No	Property	Expression
1	Dynamic viscosity	$\mu_{THNF} = \mu_{F} \phi_{1}^{ - 1} .$ $\left\{ {\phi_{1} = \left( {1 - \varpi_{1} } \right)^{2.5} \left( {1 - \varpi_{2} } \right)^{2.5} \left( {1 - \varpi_{3} } \right)^{2.5} } \right\}.$
2	Density	$\rho_{THNF} = \phi_{2} \rho_{F} .$ $\left\{ {\phi _{2} = \left( {1 - \varpi _{3} } \right)\left[ {\left( {1 - \varpi _{2} } \right)} \right.} \right.$ ${\left( {\left( {1 - \varpi _{1} } \right) + \frac{{\varpi _{1} \rho _{{s1}} }}{{\rho _{F} }}} \right)}$ $\left. {\left. { + \frac{{\varpi _{2} \rho _{{s2}} }}{{\rho _{F} }}} \right] + \frac{{\varpi _{3} \rho _{{s3}} }}{{\rho _{F} }}} \right\}$
3	Heat capacity	$\left( {\rho C_{p} } \right)_{THNF} = \phi_{4} \left( {\rho C_{p} } \right)_{F} .$ $\left\{ {\phi _{4} = \left( {1 - \varpi _{3} } \right)} \right.\left[ {\left( {1 - \varpi _{2} } \right)} \right.$ ${\left( {\left( {1 - \varpi _{1} } \right) + \frac{{\varpi _{1} \rho _{{S1}} C_{{pS1}} }}{{\rho _{F} C_{{pF}} }}} \right)}$ $\left. {\left. { + \frac{{\varpi _{2} \rho _{{S2}} C_{{pS2}} }}{{\rho _{F} C_{{pF}} }}} \right] + \frac{{\varpi _{3} \rho _{{S3}} C_{{pS3}} }}{{\rho _{F} C_{{pF}} }}} \right\}.$
4	Electrical conductivity	$\sigma_{THNF} = \sigma_{F} \phi_{3} .$ $\sigma _{{THNF}} = \sigma _{{HNF}}$ $\left[ {\frac{{\sigma _{{S3}} + 2\sigma _{{HNF}} - 2\varpi _{3} \left( {\sigma _{{HNF}} - \sigma _{{S3}} } \right)}}{{\sigma _{{S3}} + 2\sigma _{{HNF}} + \varpi _{3} \left( {\sigma _{{HNF}} - \sigma _{{S3}} } \right)}}} \right],$ $\sigma_{HNF} = \sigma_{NF} \left[ {\frac{{\sigma_{S2} + 2\sigma_{NF} - 2\varpi_{2} \left( {\sigma_{NF} - \sigma_{S2} } \right)}}{{\sigma_{S2} + 2\sigma_{NF} + \varpi_{2} \left( {\sigma_{NF} - \sigma_{S2} } \right)}}} \right],$ $\sigma_{NF} = \sigma_{F} \left[ {\frac{{\sigma_{S1} + 2\sigma_{F} - 2\varpi_{1} \left( {\sigma_{F} - \sigma_{S1} } \right)}}{{\sigma_{S1} + 2\sigma_{F} + \varpi_{1} \left( {\sigma_{F} - \sigma_{S1} } \right)}}} \right].$
5	Thermal conductivity	$k_{{THNF}} = k_{{HNF}}$ $\left[ {\frac{{k_{{S3}} + 2k_{{HNF}} - 2\varpi _{3} \left( {k_{{HNF}} - k_{{S3}} } \right)}}{{k_{{S3}} + 2k_{{HNF}} + \varpi _{3} \left( {k_{{HNF}} - k_{{S3}} } \right)}}} \right],$ $k_{{HNF}} = k_{{NF}}$ $\left[ {\frac{{k_{{S2}} + 2k_{{NF}} - 2\varpi _{2} \left( {k_{{NF}} - k_{{S2}} } \right)}}{{k_{{S2}} + 2k_{{NF}} + \varpi _{2} \left( {k_{{NF}} - k_{{S2}} } \right)}}} \right],$ $k_{{NF}} = k_{F}$ $\left[ {\frac{{k_{{S1}} + 2k_{F} - 2\varpi _{1} \left( {k_{F} - k_{{S1}} } \right)}}{{k_{{S1}} + 2k_{F} + \varpi _{1} \left( {k_{F} - k_{{S1}} } \right)}}} \right].$

Below are the equations involving skin friction, Nusselt number, and Sherwood number [27].

13 $$ C_{fx} = \frac{{\mu_{THNF} }}{{\rho_{F} u_{w}^{2} }}\left. {\frac{\partial u}{{\partial z}}} \right|_{z = 0} \Rightarrow \left( {Re_{x} } \right)^{0.5} C_{fx} = \frac{{f^{^{\prime\prime}} \left( 0 \right)}}{{\phi_{1} }}, $$

14 $$ C_{fy} = \frac{{\mu_{THNF} }}{{\rho_{F} u_{w}^{2} }}\left. {\frac{\partial v}{{\partial z}}} \right|_{z = 0} \Rightarrow \left( {Re_{x} } \right)^{0.5} C_{fy} = \frac{{h{\prime} \left( 0 \right)}}{{\phi_{1} }}, $$

15 $$ \begin{aligned} Nu_{x} = & \,\left. {\frac{ - x}{{\left( {T_{w} - T_{\infty } } \right)}}\left( {\frac{{k_{THNF} }}{{k_{F} }} + \frac{{16\sigma^{*} }}{{3k^{*} }}T_{\infty }^{3} } \right)\frac{\partial T}{{\partial z}}} \right|_{z = 0} \Rightarrow \left( {Re_{x} } \right)^{ - 0.5} \\ Nu_{x} = & \, - \left( {\frac{{k_{THNF} }}{{k_{F} }} + \frac{4}{3}k_{F} Rd} \right)\theta{\prime} \left( 0 \right), \\ \end{aligned} $$

16 $$ Sh_{x} = \left. {\frac{{ - xD_{F} }}{{D_{F} \left( {C_{w} - C_{\infty } } \right)}}\frac{\partial C}{{\partial z}}} \right|_{z = 0} \Rightarrow \left( {Re_{x} } \right)^{ - 0.5} Sh_{x} = - \chi{\prime} \left( 0 \right), $$

17 $$ {\text{where}},\,{\text{Re}}_{x} = \frac{{u_{w} x}}{{\vartheta_{F} }}\,,\,{\text{signifies}}\,\,{\text{local}}\,\,{\text{Reynolds}}\,\,{\text{number}}. $$

## Solution methodology

The governing equations of the nonlinear ODEs presented in Eqs. (8)–(11), with transformed boundary conditions in (12), are solved numerically via MATLAB bvp4c solver, which is based on the Lobatto IIIA formula, which has a higher order accuracy. The bvp4c is versatile in handling both linear and nonlinear problems. Moreover, it self-regulates the mesh (the grid points) to increase accuracy where the solution requires greater precision, thereby enhancing computational efficiency. To facilitate this approach, the system of ordinary differential equations (ODEs) and boundary conditions is transformed into a set of first-order ODEs by introducing auxiliary variables. Using the variables provided below, the MATLAB solver gives a solution using the steps given below in the flow chart.

18 $$ \left\{ \begin{gathered} f,f{\prime} ,f^{^{\prime\prime}} \hfill \\ \,\,\,\,\,h,h{\prime} \hfill \\ \,\,\,\,\,\theta ,\theta{\prime} \hfill \\ \,\,\,\,\chi ,\chi{\prime} \hfill \\ \end{gathered} \right\} \approx \left\{ \begin{gathered} f^{*} \left( 1 \right),f^{*} \left( 2 \right),f^{*} \left( 3 \right) \hfill \\ \,\,\,\,\,\,f^{*} \left( 4 \right),f^{*} \left( 5 \right) \hfill \\ \,\,\,\,\,\,f^{*} \left( 6 \right),f^{*} \left( 7 \right) \hfill \\ \,\,\,\,\,\,f^{*} \left( 8 \right),f^{*} \left( 9 \right) \hfill \\ \end{gathered} \right\}. $$

Then the equations take the form,

19 $$ \begin{aligned} f^{^{\prime}\,^{\prime}\,^{\prime}} = & \,\lambda \left( {f^{*} \left( 2 \right) - S} \right) - \phi_{1} \phi_{2} \left[ {2Rpf^{*} \left( 4 \right) + S^{2} + f^{*} \left( 1 \right)f^{*} \left( 3 \right) - \left[ {f^{*} \left( 2 \right)} \right]^{2} + Fr\left( {S^{2} - \left( {f^{*} \left( 2 \right)} \right)^{2} } \right)} \right] \\ & \, + M\left( {f^{*} \left( 2 \right) - S} \right)\phi_{1} \phi_{3} , \\ \end{aligned} $$

20 $$ \begin{aligned} h^{^{\prime}\,^{\prime}} = & \,\lambda f^{*} \left( 4 \right) + \phi_{1} \phi_{2} \left[ {2Rpf^{*} \left( 5 \right) - f^{*} \left( 1 \right)f^{*} \left( 5 \right) + f^{*} \left( 2 \right)f^{*} \left( 4 \right) + Fr\left( {f^{*} \left( 4 \right)} \right)^{2} } \right] \\ & \, + Mf^{*} \left( 4 \right)\phi_{1} \phi_{3} , \\ \end{aligned} $$

21 $$ \begin{aligned} \theta^{\,^{\prime}\,^{\prime}} = & \,\left( { - \frac{1}{{\frac{{k_{THNF} }}{{k_{F} }} + \frac{4}{3}Rd}}} \right)\left( {Pr} \right)\left[ {Rr\lambda^{*} exp\left( { - \frac{E}{{\left( {\delta f^{*} \left( 6 \right) + 1} \right)}}} \right)\left( {\delta f^{*} \left( 6 \right) + 1} \right)^{n} f^{*} \left( 8 \right)} \right] \\ & \, + f^{*} \left( 7 \right)\phi_{4} f^{*} \left( 1 \right), \\ \end{aligned} $$

22 $$ \chi^{\prime \prime } = - Sc\left[ {f^{*} \left( 1 \right)f^{*} \left( 9 \right) - Rr\lambda^{*} epx\left( { - \frac{E}{{\left( {\delta f^{*} \left( 6 \right) + 1} \right)}}} \right)\left( {\delta f^{*} \left( 6 \right) + 1} \right)^{n} f^{*} \left( 8 \right)} \right]. $$

The BCs are,

23 $$ \left. {\begin{array}{*{20}l} {\left( {f^{*} \left( 1 \right),f^{*} \left( 2 \right),f^{*} \left( 4 \right),f^{*} \left( 6 \right),f^{*} \left( 8 \right)} \right)_{\eta = 0} = \left( {0,1,0,1,1} \right),} \hfill \\ {\left( {f^{*} \left( 2 \right),f^{*} \left( 4 \right),f^{*} \left( 6 \right),f^{*} \left( 8 \right)} \right)_{\eta \to \infty } = \left( {S,0,0,0} \right).} \hfill \\ \end{array} } \right\} $$

**Fig. 2:**
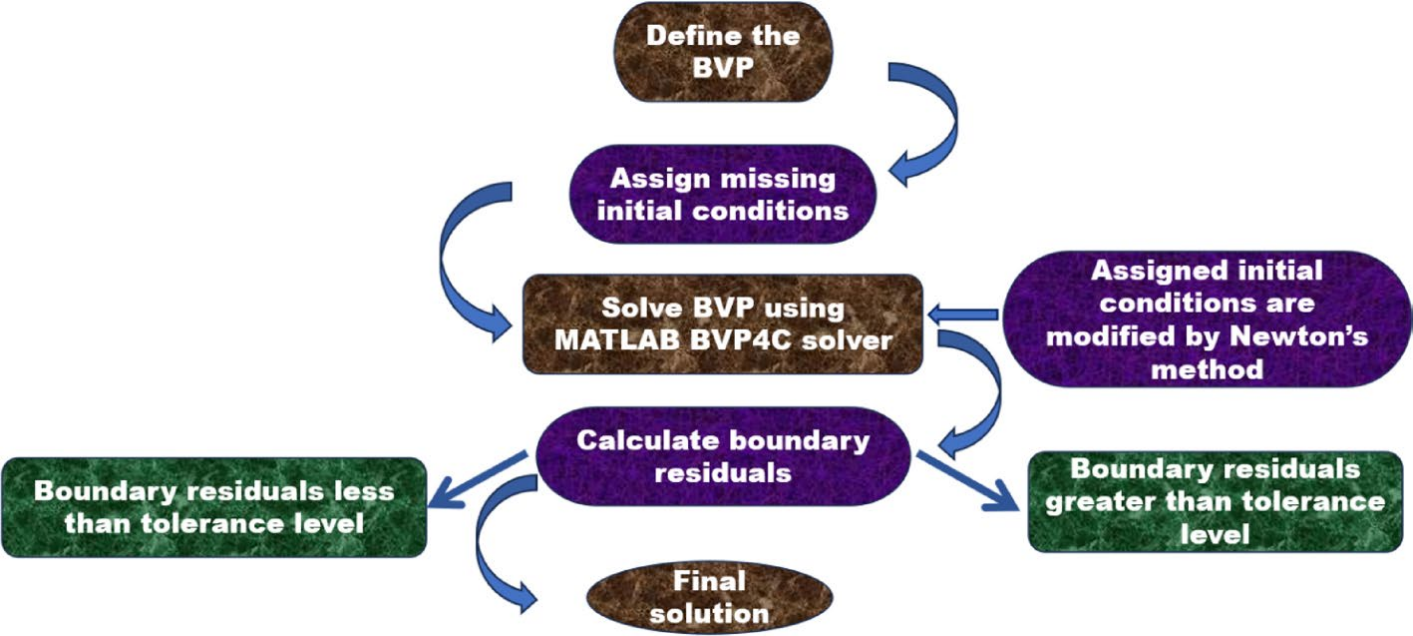
BVP4c procedure flow chart

This procedure is repeated until the convergence of results attains the desired degree of accuracy ${10}^{-6}$. We must select a finite value for the boundary as $\eta \to \infty$, which we have chosen as 7. The flowchart for the bvp4c is given in Fig. 2 and Table 3.

**Table 3:** Numerical values of Physical quantities used in our research work [8, 29]

Thermophysical property	Base fluid C_2_H_6_O_2_ + H_2_O	CuO	Nanoparticles TiO_2_	Ag
Density $\left( \rho \right)\left[ {kgm^{ - 3} } \right]$	1056	6320	4250	10,490
Specific Heat $\left( {C_{P} } \right)\left[ {J\left( {Kkg} \right)^{ - 1} } \right]$	3288	531.8	686.2	235
Electrical Conductivity $\left( \sigma \right)\left[ {Sm^{ - 1} } \right]$	0.00509	$6.9 \times 10^{ - 2}$	$3.5 \times 10^{6}$	$6.3 \times 10^{7}$
Thermal Conductivity $\left( k \right)\left[ {W\left( {Km} \right)^{ - 1} } \right]$	0.425	76.5	8.9538	429

## Results and discussion

**Fig. 3:**
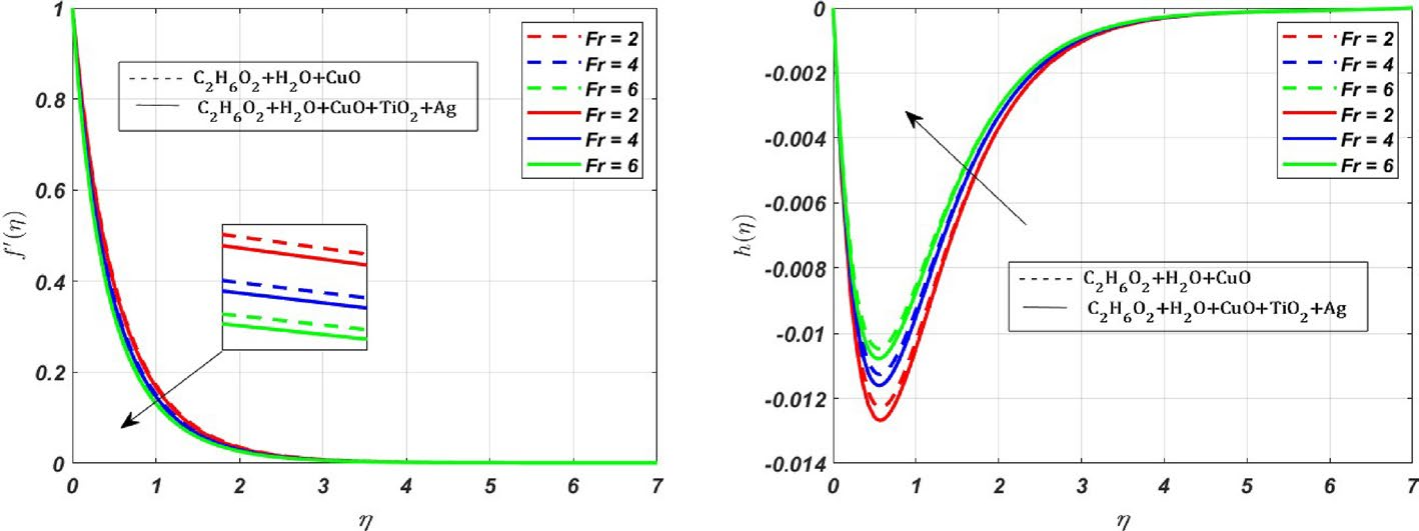
Nature of ${f}{\prime}(\eta )$ and $h$ for $Fr=\text{2,4},6$

**Fig. 4:**
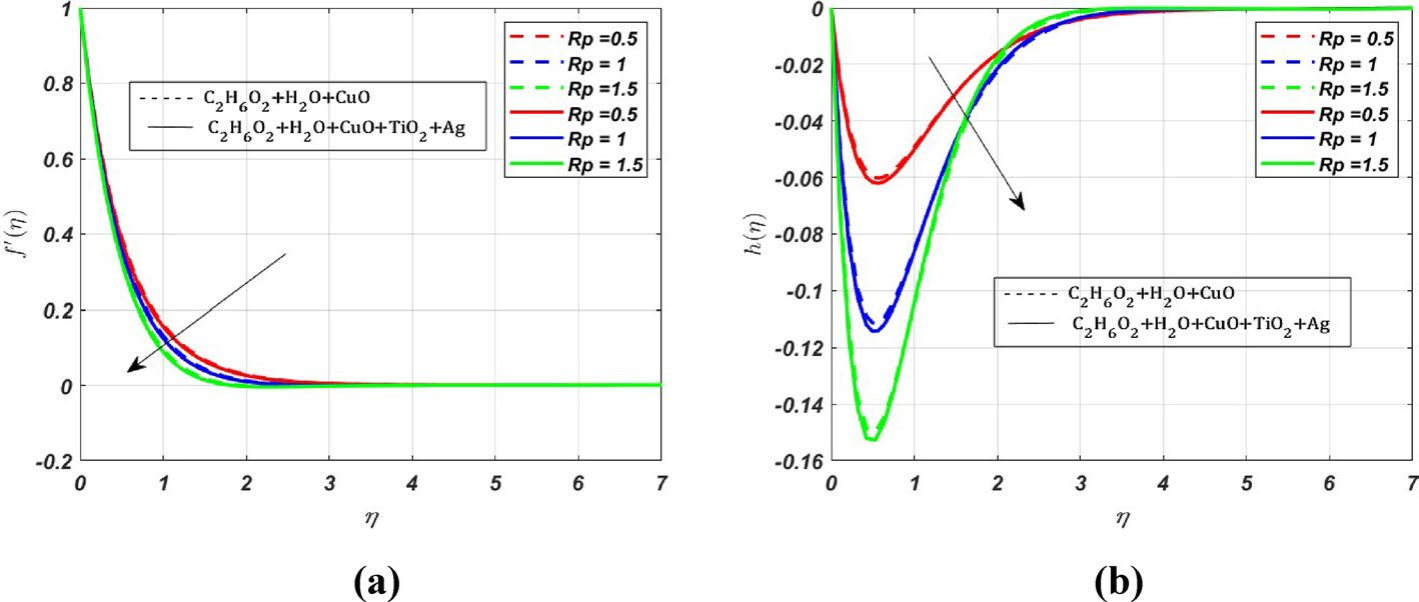
Nature of ${f}{\prime}(\eta )$ and $h$ for $Rp=0.5, 1, 1.5$

**Fig. 5:**
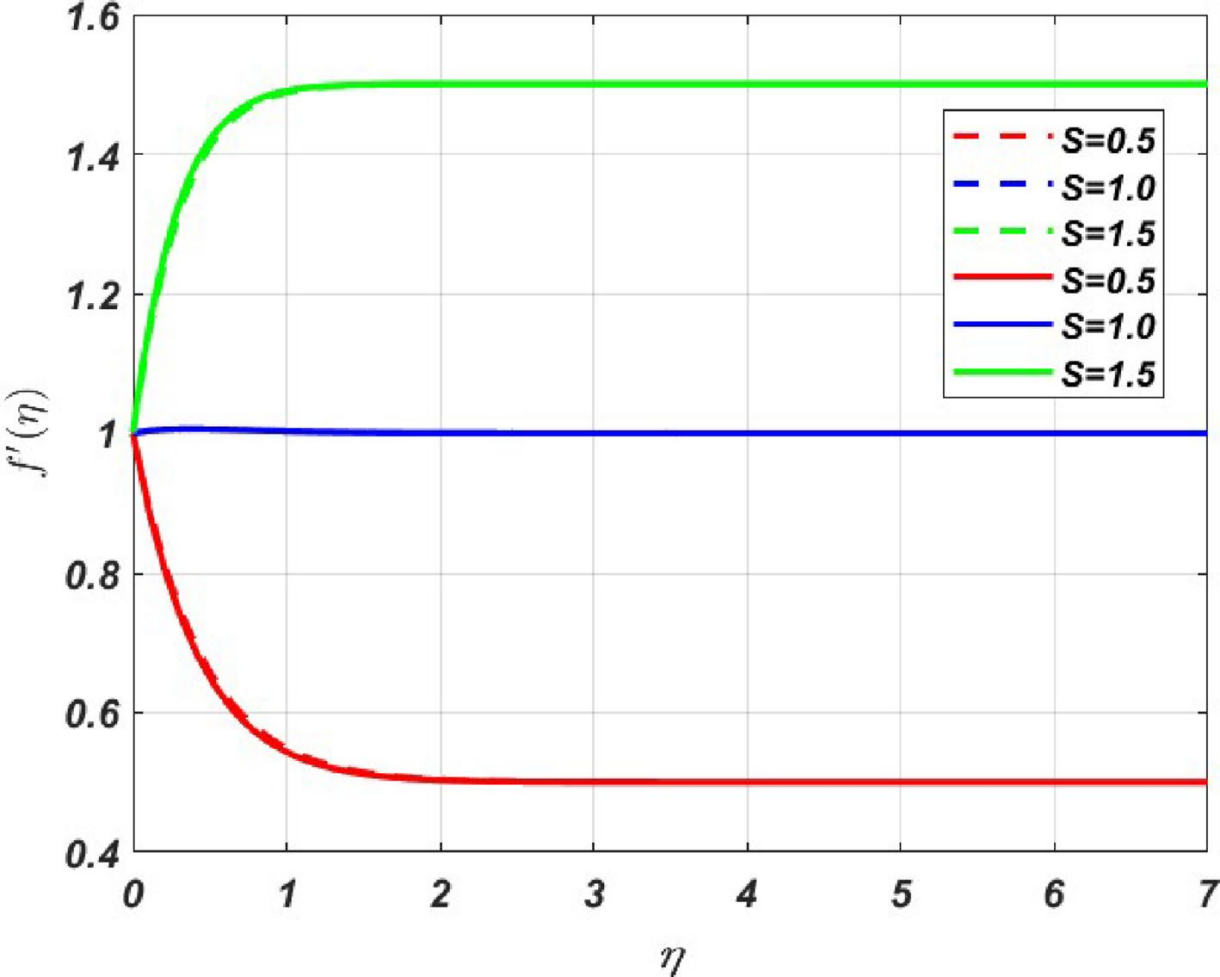
Nature of ${f}{\prime}(\eta )$ for *S* = 0.5, 1, 1.5

**Fig. 6:**
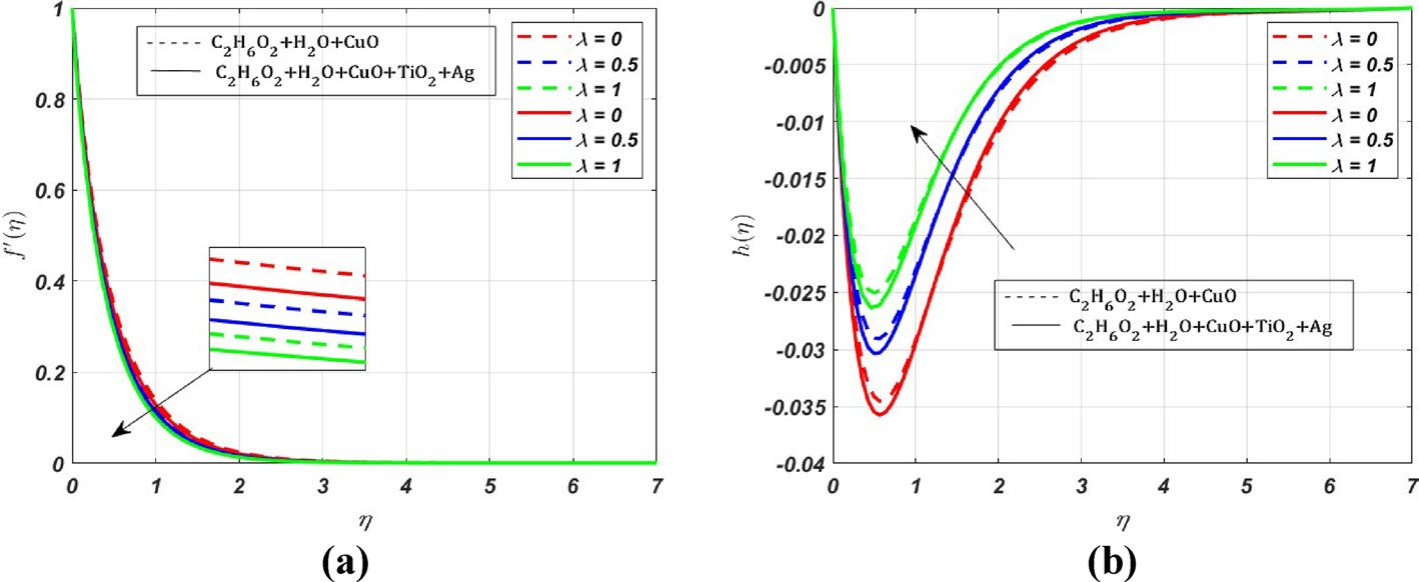
Nature of ${f}{\prime}(\eta )$ and $h$ for $\lambda =0, 0.5, 1$

**Fig. 7:**
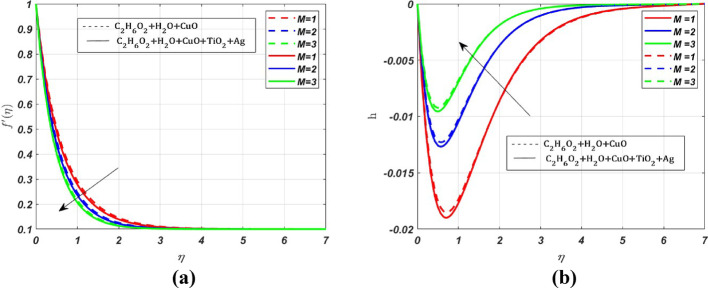
Nature of ${f}{\prime}(\eta )$ and $h$ for $M=1, 2, 3$

**Fig. 8:**
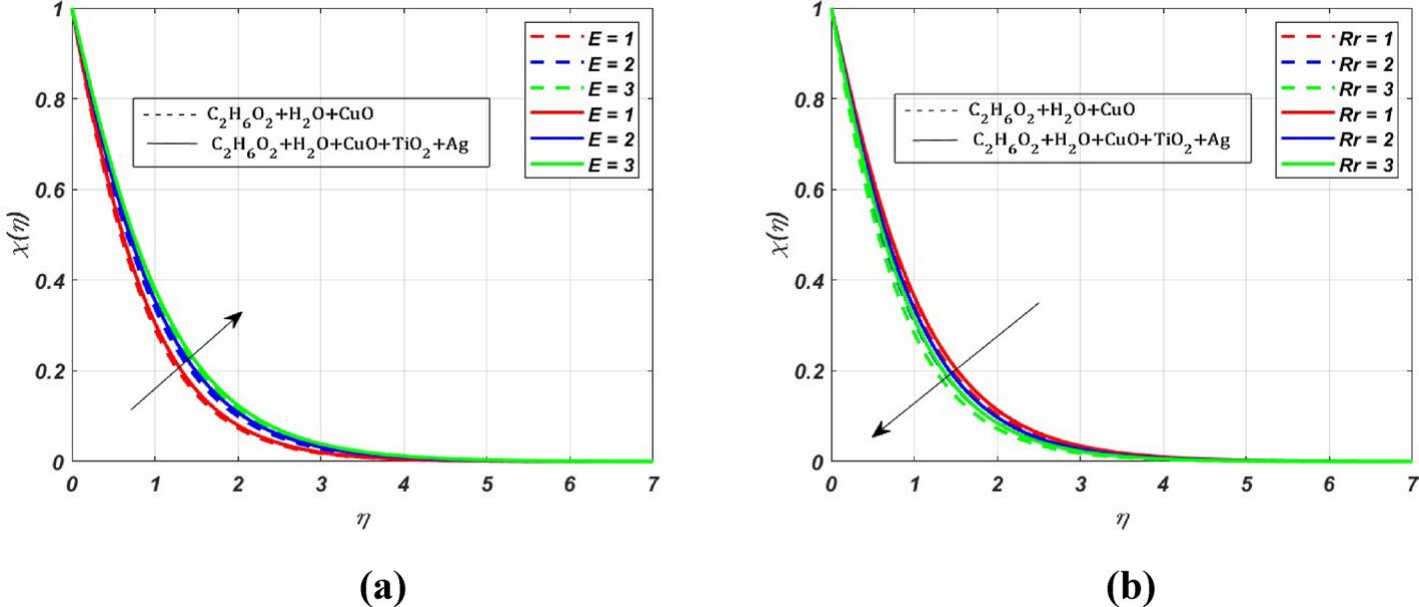
Nature of $\chi (\eta )$ for $E=Rr=1, 2, 3$

**Fig. 9:**
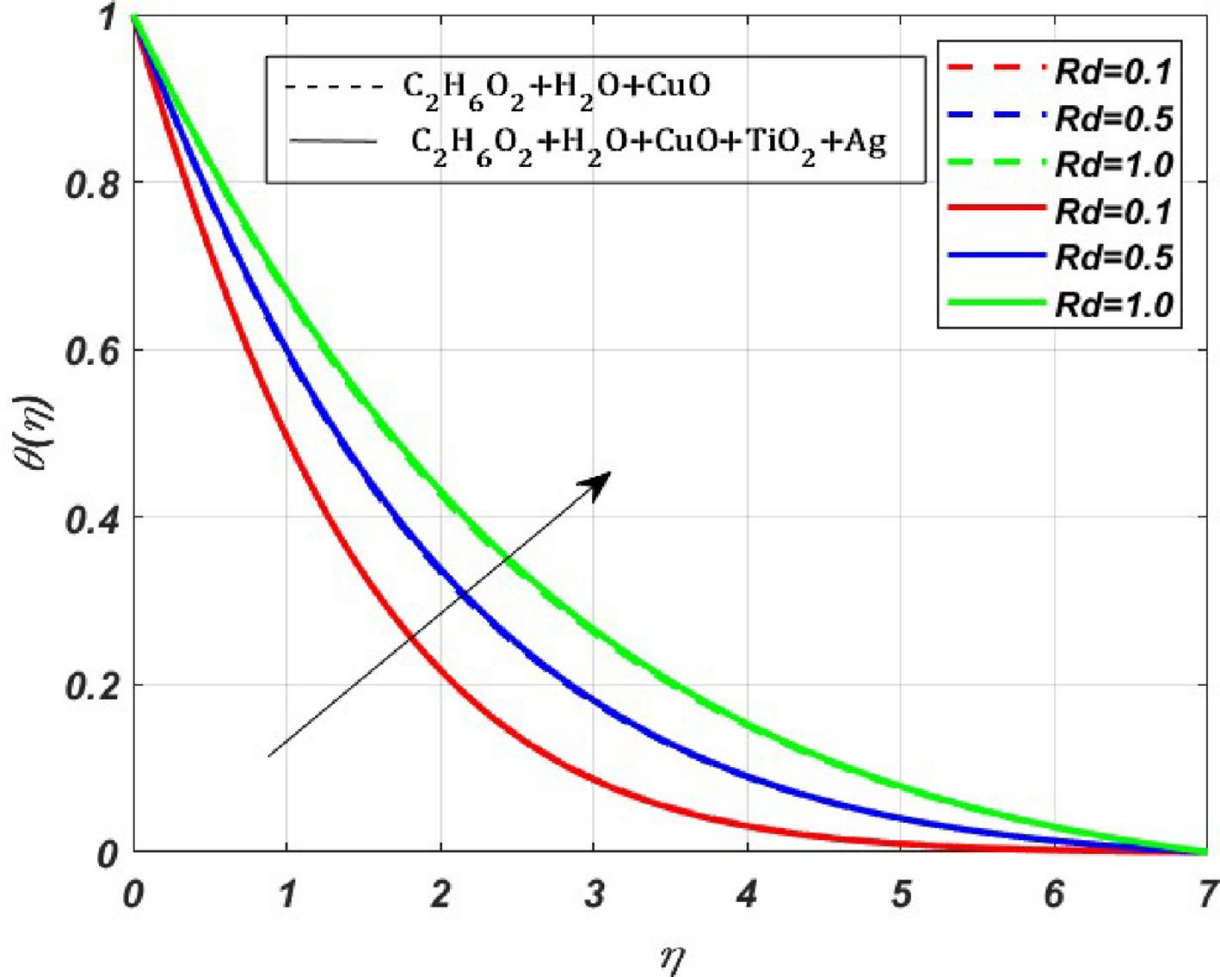
Nature of $\theta (\eta )$ for $Rd=0.1, 0.5, 1$

**Fig. 10:**
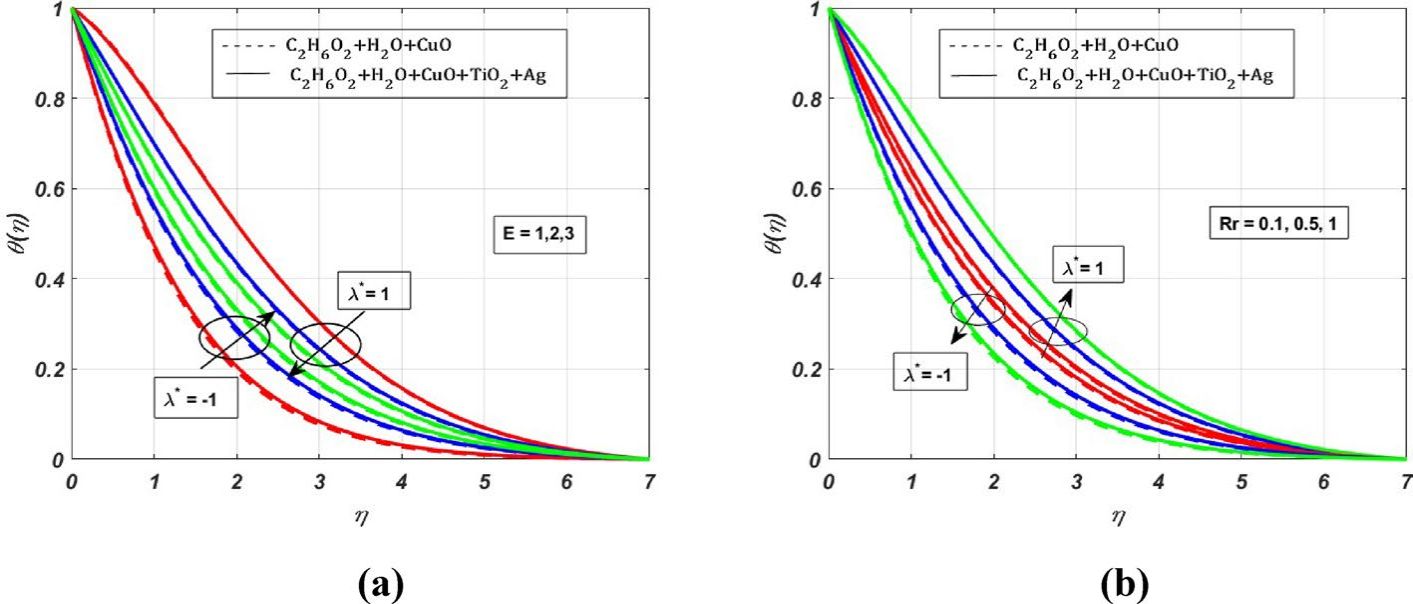
Nature of $\theta (\eta )$ for $E=1, 2, 3$ and $Rr=0.1, 0.5, 1$ when ${\lambda }^{*}=\pm 1$

**Fig. 11:**
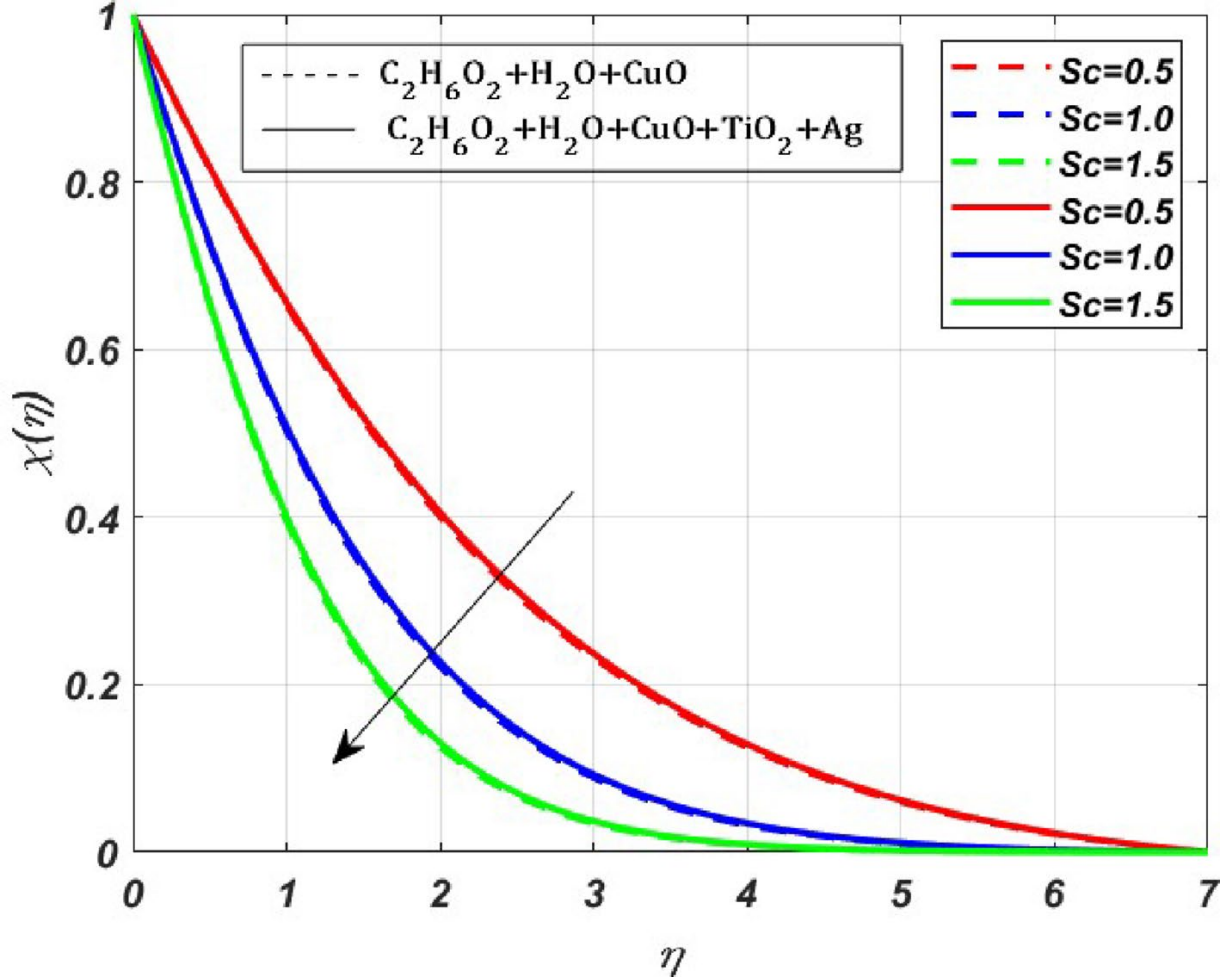
Nature of $\chi (\eta )$ for $Sc=0.5, 1, 1.5$

MATLAB bvp4c solver is used to interpret the solution in the form of graphs with various parameters. The effect of various parameters on the primary velocity ${({\varvec{f}}}^{\boldsymbol{^{\prime}}}({\varvec{\eta}}))$, secondary velocity $({\varvec{h}}\left({\varvec{\eta}}\right))$, temperature $({\varvec{\theta}}({\varvec{\eta}}))$, concentration $({\varvec{\chi}}({\varvec{\eta}}))$ profiles and skin friction, Nusselt number and Sherwood number are represented in Figs. 3, 4, 5, 6, 7, 8, 9, 10, 11. The porous medium’s inertial barrier to fluid flow increases with the Darcy–Forchheimer parameter. As seen in Fig. 3(a), the fluid encounters drag as a result of this increased resistance, which lowers the primary velocity. However, as Forchheimer values increase, the secondary velocity increases as well. In Fig. 3(b), the Coriolis force causes ${{\text{H}}_{2}\text{O}-\text{ C}}_{2}{\text{H}}_{6}{\text{O}}_{2}+\text{CuO}+{\text{TiO}}_{2}+\text{Ag}$ to grow more intensely than ${{\text{H}}_{2}\text{O}-\text{C}}_{2}{\text{H}}_{6}{\text{O}}_{2}+\text{CuO}$. Whenever a fluid is in a rotating frame of reference, it experiences a fictitious force known as the Coriolis force. This force acts along the axis of rotation; for increasing values of the rotation parameter, it decreases the fluid motion in primary and secondary directions; hence, we can see a decrease in both the fluid velocities in Fig. 4a and b. The rate of stretching or contracting of the surface is indicated by the stretching parameter. Figure 5 illustrates how the velocity profile is affected by the stretching parameter $({\varvec{S}})$. When the stretching parameter $S>1$, the surface stretches due to which the fluid flows easily through the surface, but when $S<1$, the surface contracts by reducing the velocity of the fluid. In contrast, when $S=1$, the surface remains unchanged. By accelerating the fluid flow close to the stretching surface, a stretching parameter essentially serves as a driving factor, resulting in higher velocities, thereby increasing the transport phenomena. Figure 6a illustrates the impact of the porosity parameter $({\varvec{\lambda}})$ on the primary velocity profile ${f}{\prime}\left(\eta \right)$. In a porous medium, higher porosity implies more open space, which acts as a barrier to fluid flow. The fluid has to navigate through a complex network of pores, resulting in increased resistance, which decreases the velocity profile by **5%** when $\lambda$ varies from 0 to 0.5. But the *h* profile increases by **8%** with increasing porosity parameter for both nanofluid and ternary fluid, due to the combined influence of Coriolis and porosity parameter, as illustrated in Fig. 6b. When an electrically conducting fluid moves through a magnetic field (*M*), a current is induced within the fluid due to electromagnetic induction. This induced current interacts with the applied field, producing a force (Lorentz force) that opposes the fluid’s motion. Therefore, by increasing *M* values, the Lorentz force also increases, which opposes the fluid’s motion, resulting in the reduction of the fluid velocity as shown in Fig. 7a, whereas Fig. 7b shows an opposite behaviour due to the electromagnetic coupling caused by the increased magnitude of *M* that tends to increase the *h* profile.

The change in *M* values has significantly increased the ${\varvec{\theta}}({\varvec{\eta}})$ profile of the ternary nanofluid by **1.2%** when compared to the nanofluid. The impact of the activation energy parameter $({\varvec{E}})$, and the reaction rate parameter $\left({\varvec{R}}{\varvec{r}}\right)$ on the concentration profile is depicted in Fig. 8. We can observe a rise in the concentration profile due to the activation energy parameter. The rise in concentration can be attributed to increased collisions between reactant molecules, resulting from higher activation energy, which acts as a booster to the concentration profile. Whereas there is an opposite behaviour in the concentration profile due to the reaction rate parameter, as seen in Fig. 8b.

The effect of the radiation parameter (***Rd***) on $\theta (\eta )$ is displayed in Fig. 9. Whenever we increase the radiation parameter **(0.1–0.5)**, the temperature profile rises by **1.09%,** this increase is mainly due to the increased radiation absorption by the fluid particles. Therefore, we can see the growth of the temperature profile. The effect of endothermic $({\lambda }^{*}>0)$ and exothermic reactions $({\lambda }^{*}<0)$ on the activation energy parameter (***E***) and reaction rate (***Rr***) parameter are presented in Fig. 10a and b. The activation energy parameter and reaction rate parameter are intricately linked. Notably, for increasing values of* E* in an endothermic reaction $({\lambda }^{*}>0)$, a decrease in $\theta (\eta )$ is **4%**, whereas the $\theta (\eta )$ profile increases by **0.5%** for increasing *Rr* values. This decrease is due to the absorption of energy from the surroundings to carry out the reaction, which tends to decrease the $\theta$ profile. Conversely, for growing *Rr* values, the reactant molecules have enough energy to overcome the activation energy barrier, leading to a faster reaction rate. For the exothermic case $\left({\lambda }^{*}<0\right)$, there is a visible rise in the temperature profile by **1%** due to the increased magnitude of *E*. As the reaction is exothermic, the released energy contributes to the elevation of $\theta (\eta )$, whereas $\theta$ profile depletes by **0.2%** for growing *Rr* values. The benefit of this increased heat transfer is evident in heat exchangers, petroleum and chemical processing, advanced heat transfer in rotating machinery, and biomedical and pharmaceutical processing industries. The effect due to the Schmidt parameter $({\varvec{S}}{\varvec{c}})$ on the concentration profile $(\chi (\eta ))$ is presented in Fig. 11. The concentration profile exhibits depletion due to the diminished mass transfer rate, which occurs when the Schmidt number increases.

**Fig. 12:**
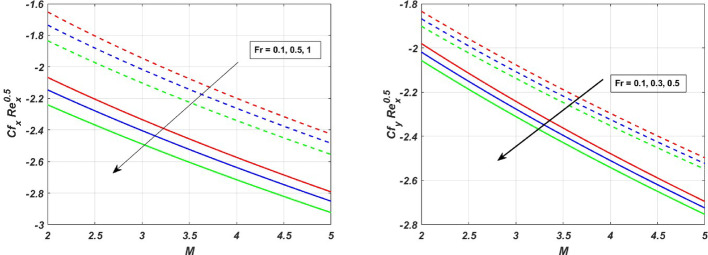
Variation of primary and secondary skin friction coefficient ${{\varvec{C}}}_{{\varvec{f}}{\varvec{x}}}$ and ${{\varvec{C}}}_{{\varvec{f}}{\varvec{y}}}$ on $M$ for a change in $Fr$

**Fig. 13:**
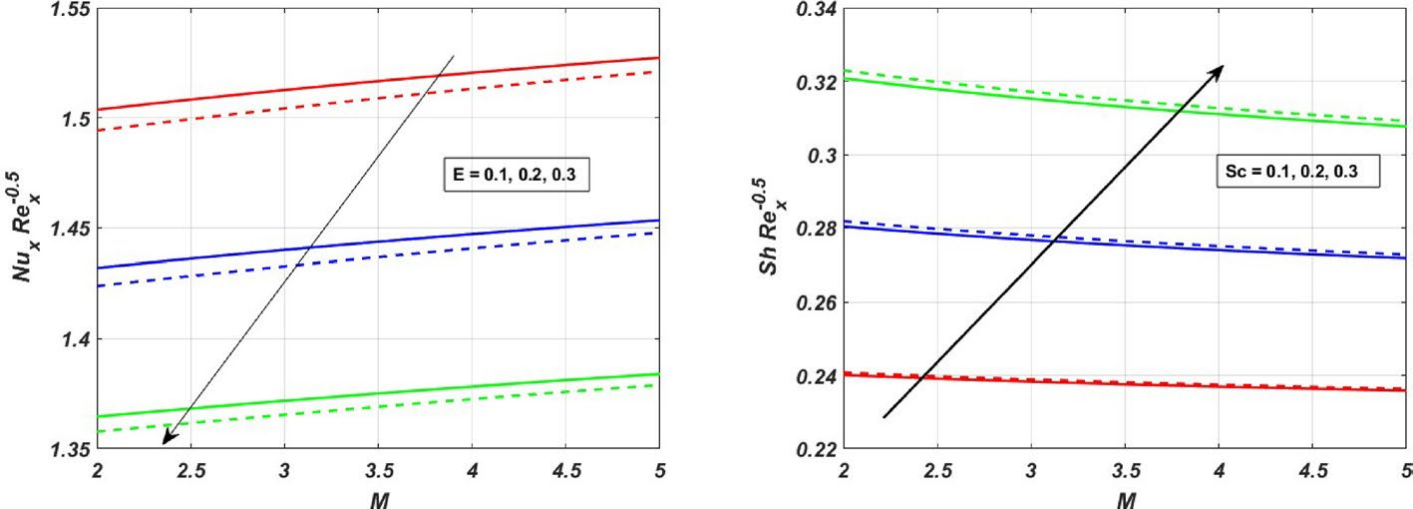
Variation in the heat and mass transfer rates ${{\varvec{N}}{\varvec{u}}}_{{\varvec{x}}}$ and ${{\varvec{S}}{\varvec{h}}}_{{\varvec{x}}}$ on $M$ for a change in *E* and $Sc$ values

**Fig. 14:**
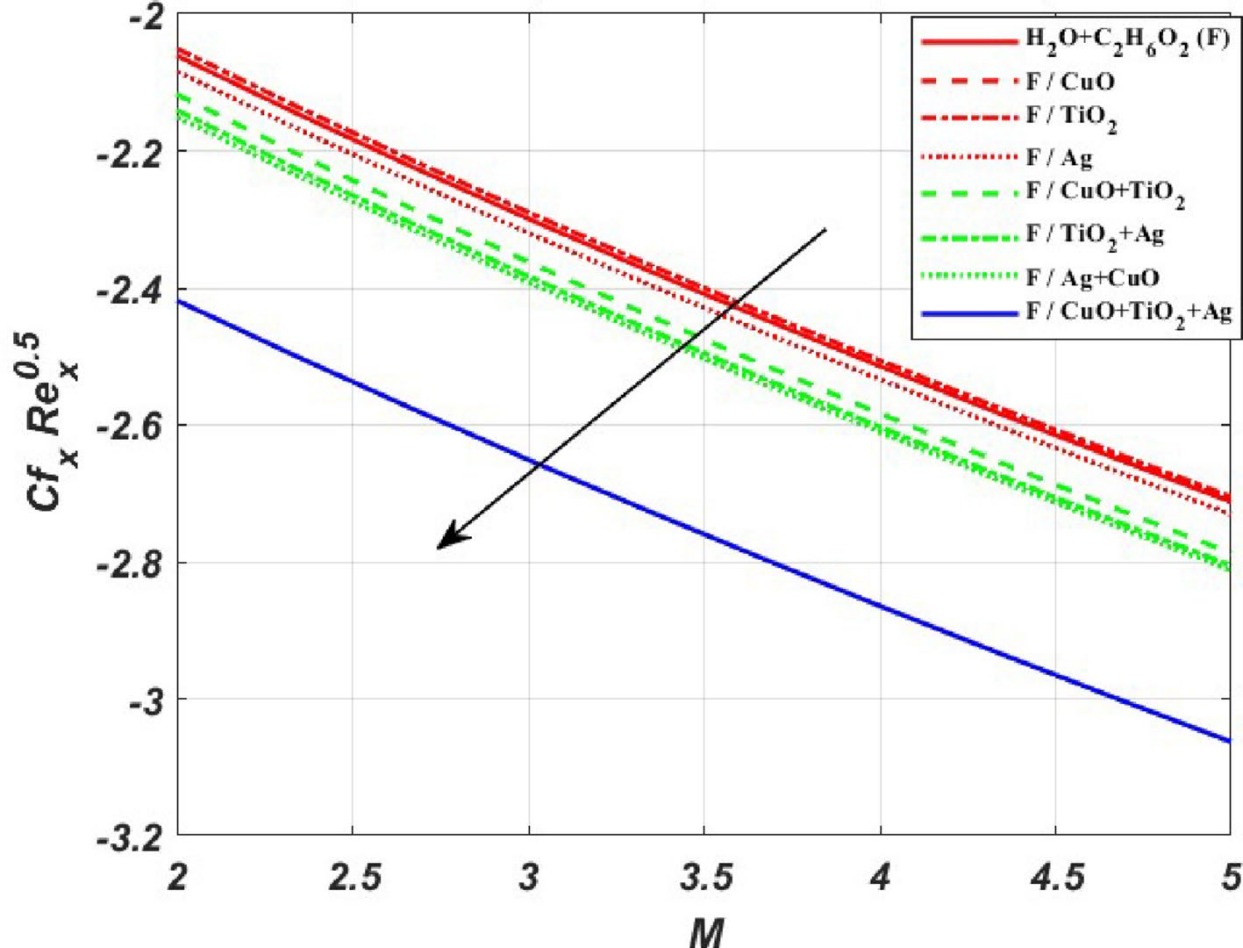
The comparison of skin friction coefficient for nanofluid, hybrid nanofluid and ternary hybrid nanofluid on *M* for changing *Rp* values

**Fig. 15:**
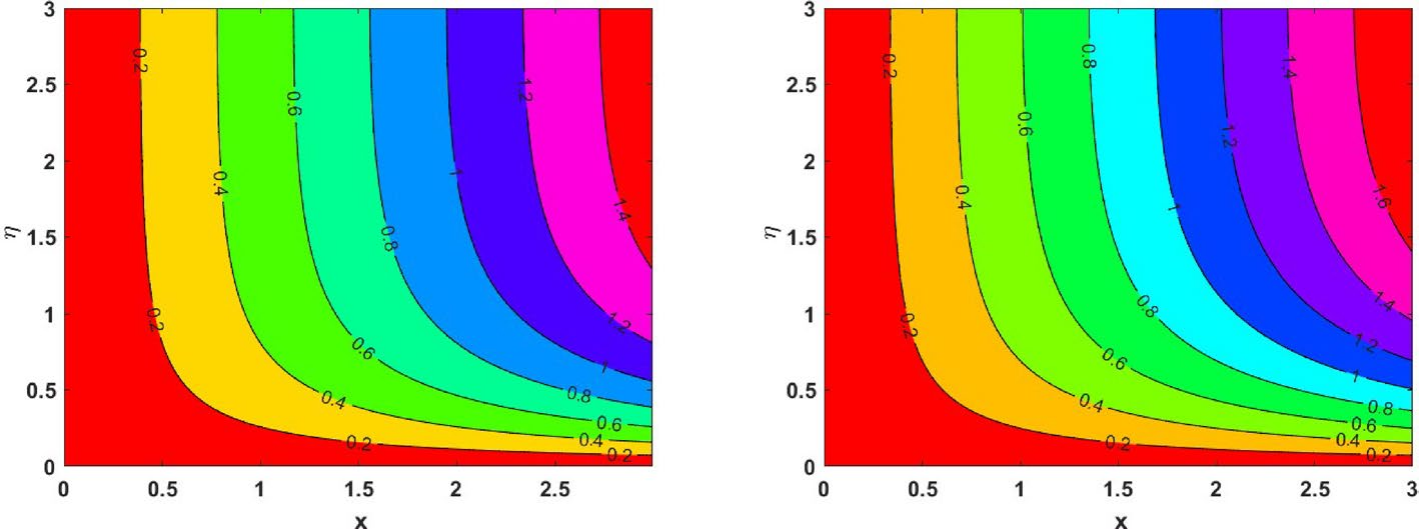
Streamlines in the absence of ${\varvec{\lambda}}$ and in the presence of ${\varvec{\lambda}}$

**Fig. 16:**
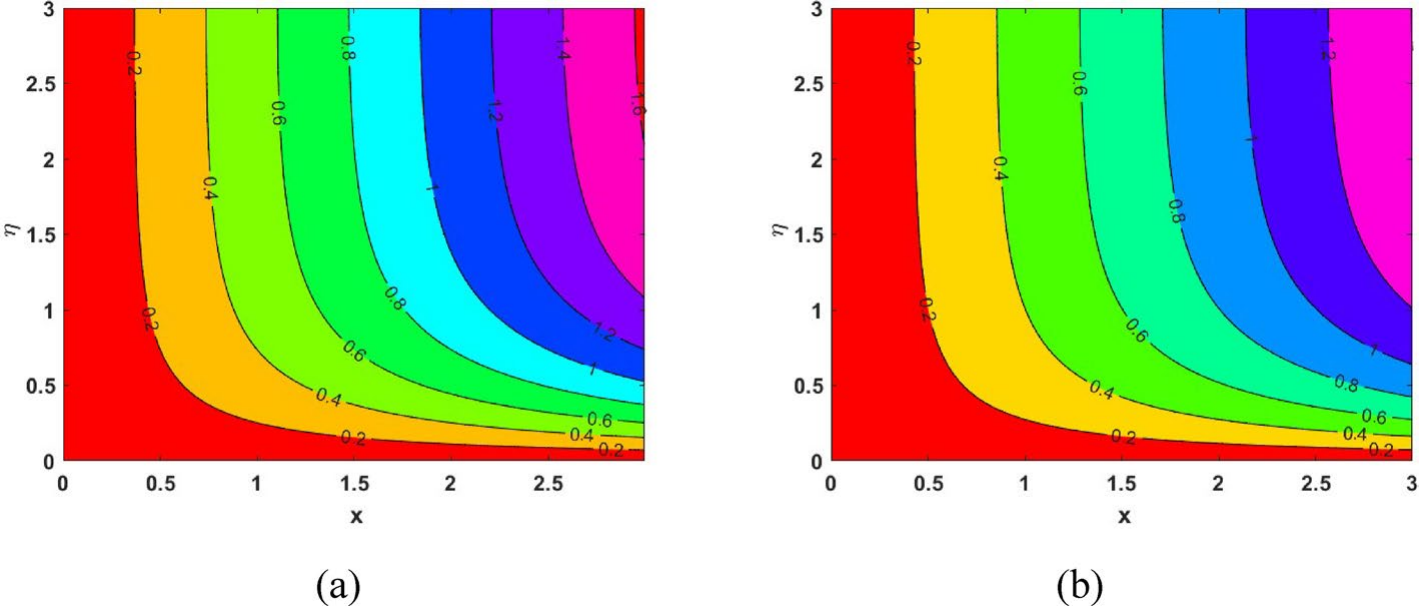
Streamlines for ***Fr* = *1*** and ***Fr* = *5***

**Fig. 17:**
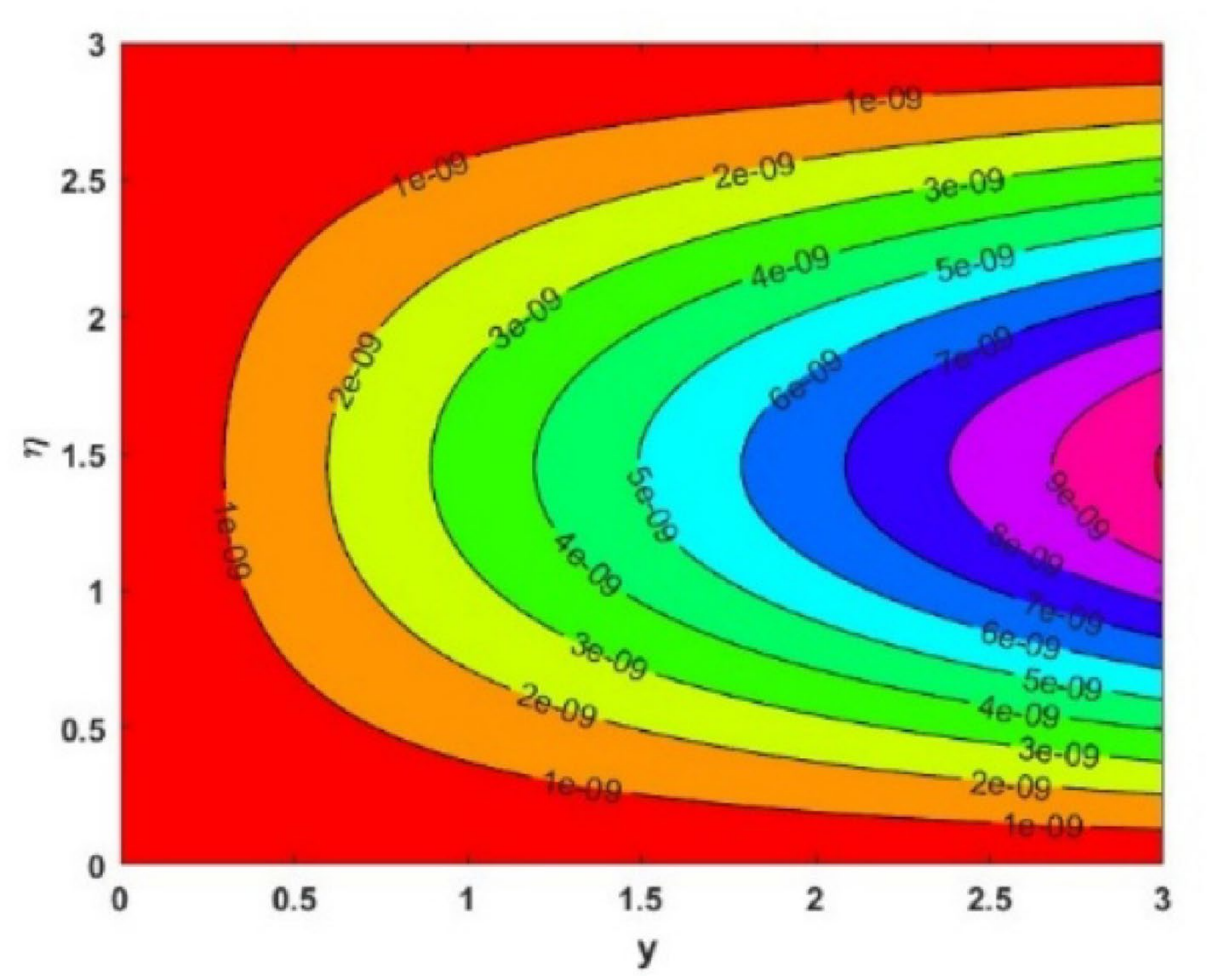
Streamlines for secondary direction

**Fig. 18:**
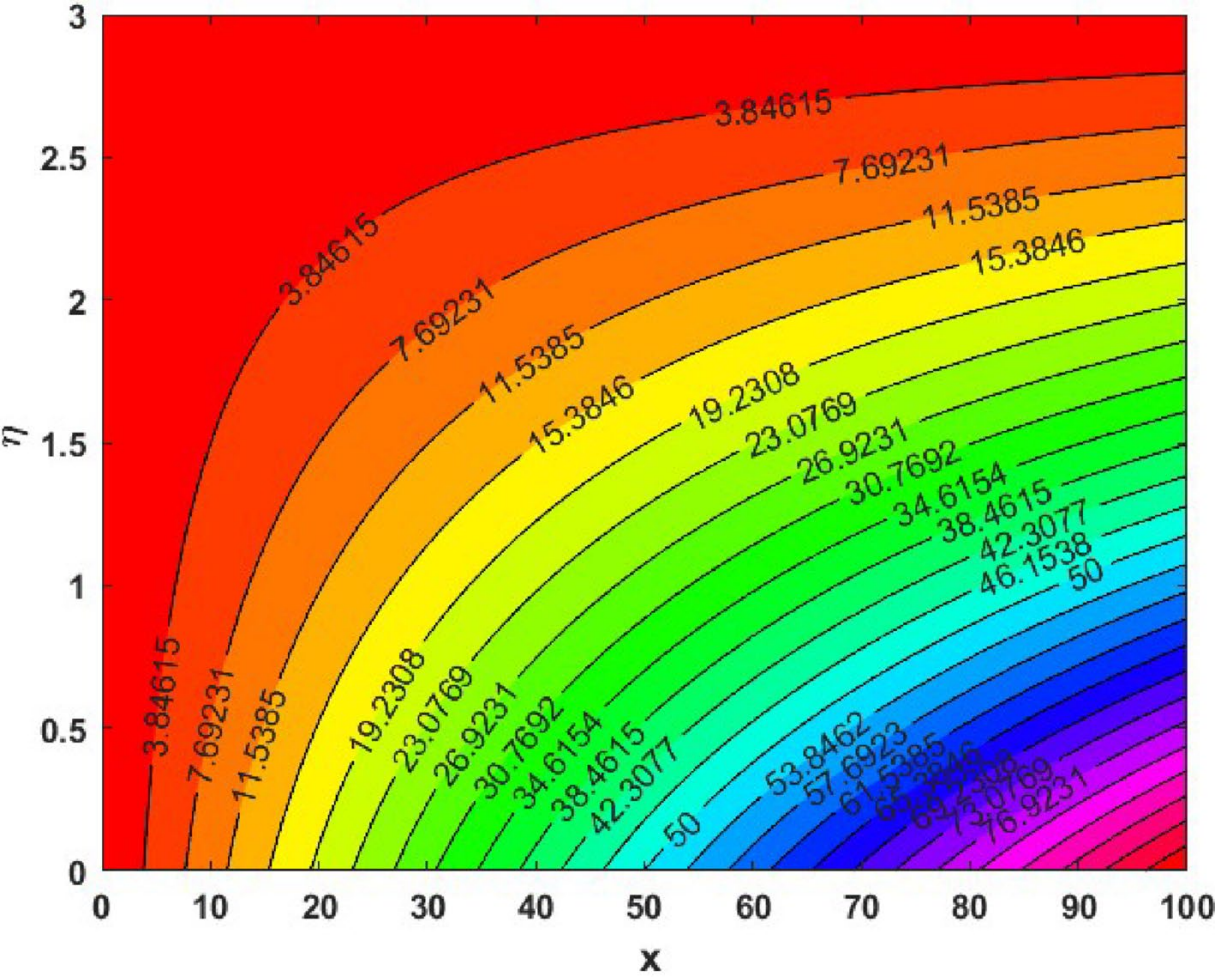
Isotherm patterns

The influence of the magnetic field strength (*M*) on the primary skin friction coefficient (${{\varvec{C}}}_{{\varvec{f}}{\varvec{x}}}$) and secondary skin friction coefficient (${{\varvec{C}}}_{{\varvec{f}}{\varvec{y}}}$) is examined for varying values of the Forchheimer number (*Fr*), as illustrated in Fig. 12a and b, we can see a diminishing behaviour of the skin friction profiles for increasing *Fr* values in the presence of the magnetic field. Figure 13a and b depict the variations of heat and mass transfer coefficients, respectively. The heat transfer coefficient ${{\varvec{N}}{\varvec{u}}}_{{\varvec{x}}}$ undergoes depletion on increasing *E* values. But there is an increase in the mass transfer coefficient ${{\varvec{S}}{\varvec{h}}}_{{\varvec{x}}}$, due to enhanced mass diffusivity, which is caused by increasing the Schmidt parameter. Figure 14 represents the presence of mono, bi and tri nanoparticles in the base fluid. This figure shows that there is a drastic decrease in the skin friction coefficient for the ternary nanofluid compared to the other two. Figures 15 and 16 represent the streamlines for ${\varvec{\lambda}}$ and ***Fr***. In Fig. 15**,** we can observe enlarged streamlines, which represent higher velocity regions in the absence of ${\varvec{\lambda}}$, and less-wider streamlines represent low velocity regions in the presence of ${\varvec{\lambda}}$, respectively. Whereas Fig. 16 shows a difference in the streamline pattern due to growing ***Fr*** values. Figure 17 shows the streamlines in the y-direction, and Fig. 18 shows the pattern of the isothermal lines. Table 4 describes the validation of the present work. The variations in primary and secondary skin friction coefficients, Nusselt and Sherwood numbers are presented in Tables 5 and 6.

**Table 4:** Validation of the present work for ${-f}^{{\prime}{\prime}}\left(0\right) \& {-g}{\prime}\left(0\right)$ in the absence of $S=M=Fr=\lambda {=\varpi }_{1}{=\varpi }_{2}{=\varpi }_{3}=0$ for *Rp* values

*Rp*		0	0.5	1
Wang [13]	${-f}^{{\prime}{\prime}}\left(0\right)$	1	1.1384	1.3250
Madiwal et al. [27]	${-f}^{{\prime}{\prime}}\left(0\right)$	1	1.138591	1.325162
Present Study	${-f}^{{\prime}{\prime}}\left(0\right)$	1	1.138365	1.325030
Wang [13]	${-g}{\prime}\left(0\right)$	0	0.5128	0.8371
Shah and Awan [28]	${-g}{\prime}\left(0\right)$	0	0.512762	0.837098
Present study	${-g}{\prime}\left(0\right)$	0	0.512729	0.837098

**Table 5:** Computational values for primary and secondary skin friction coefficients of mono and ternary nanofluids for different parameter values

*Rp*	*S*	*M*	*Fr*	*λ*	$Re_{x}^{0.5} C_{fx}$ Mono case	$Re_{x}^{0.5} C_{fx}$ Ternary case	$Re_{x}^{0.5} C_{fy}$ Mono case	$- Re_{x}^{0.5} C_{fy}$ Ternary case
0.1	0	2	2	0.5	− 2.2649	− 2.4193	− 0.0558	− 0.0614
0.5					− 2.2819	− 2.4384	− 0.2775	− 0.3053
1.0					− 2.3304	− 2.4932	− 0.5414	− 0.5944
	0				− 2.2663	− 2.421	− 0.0558	− 0.0613
	0.5				− 1.361	− 1.4593	− 0.0874	− 0.0964
	1				− 0.0019	− 0.0021	− 0.1214	− 0.1339
		1			− 2.0184	− 2.1619	− 0.0664	− 0.0729
		2			− 2.2649	− 2.4193	− 0.0558	− 0.0614
		3			− 2.4870	− 2.6516	− 0.0491	− 0.0541
			2		− 2.2649	− 2.4193	− 0.0558	− 0.0614
			4		− 2.5603	− 2.7421	− 0.0534	− 0.0587
			6		− 2.8254	− 3.0312	− 0.0514	− 0.0565
				0.5	− 2.1472	− 2.2959	− 0.0604	− 0.0663
				1	− 2.2649	− 2.4193	− 0.0558	− 0.0614
				1.5	− 2.3782	− 2.5366	− 0.0522	− 0.0575

**Table 6:** Computational values for Nusselt (${Nu}_{x}$) and Sherwood (${Sh}_{x}$) numbers are presented for mono and ternary nanofluids, exploring the effects of various parameter values

Rp	S	λ	Rd	Sc	Rr		E		Nu_x_%	Sh_x_%
0.1	0.001	0.5	0.1	0.8	0.5		2		8.84	5.52
0.5									9.12	5.71
1									9.86	6.23
	0								8.87	5.53
	0.5								1.91	2.72
	1								0.28	1.79
		0							8.34	5.29
		0.5							8.84	5.52
		1							9.22	5.74
			0.1						3.92	0.24
			0.5						2.79	0.36
			1						1.84	0.36
				0.5					3.81	0.38
				1					3.86	0.46
				1.5					3.90	0.47
						1			32.23	0.32
					λ^*^ = + 1	2			7.97	0.19
						3			6.94	0.13
						1			5.10	0.34
					λ^*^ = − 1	2			5.23	0.22
						3			5.30	0.16
								1	12.49	0.28
							λ^*^ = + 1	2	1.08	0.44
								3	3.33	0.57
								1	5.13	0.29
							λ^*^ = − 1	2	4.81	0.45
								3	4.48	0.57
									6.65	1.86

To calculate the ${Nu}_{x}\%$ and ${Sh}_{x}\%$ over different parameter values for the ternary hybrid nanofluid and the nanofluid [31]. The following formula calculates the percentage increase in heat and mass transfer during fluid flow. The obtained values are taken in absolute terms (modulus) for each parameter value, providing a quantitative measure of the enhancement.

$$ Nu_{x} \% = \left| {\frac{{Nu_{THNF} - Nu_{NF} }}{{Nu_{NF} }}} \right| \times 100,\,\,\,and\,\,Sh_{x} \% = \left| {\frac{{Sh_{THNF} - Sh_{NF} }}{{Sh_{NF} }}} \right| \times 100 $$

This formula calculates the percentage increase in heat and mass transfer during fluid flow. The obtained values are taken in absolute terms (modulus) for each parameter value, providing a quantitative measure of the enhancement.

## Conclusion

The present study investigates the significance of a magnetized ternary hybrid nanofluid, incorporating stagnation point, Darcy–Forchheimer, and endothermic/exothermic chemical reactions through a porous medium on a rotating stretching sheet. The study’s findings are highly significant for cooling of systems, lubrication, antibacterial activity, and enhanced heat transfer in industries. Following a thorough computational evaluation of our current work, we can say,

- The implementation of the $\text{CuO}+{\text{TiO}}_{2}+\text{Ag}$ nanoparticles into the base fluid water-ethylene glycol has profoundly elevated the thermal efficiency by **6.65%** when compared with the nanofluid.
- The effect of Darcy–Forchheimer and Magnetic parameter is significant, due to which there is depletion in the primary velocity profiles. In contrast, secondary profiles experience a rise due to the growing magnitude of *Fr* and *M*.
- The growing magnitude of *M* and *Fr* values increases the temperature profile by **1.2%** and **0.85%** respectively.
- The thermal profile increases for increasing values of *Rr* by **0.5%** in the exothermic case and decreases by **0.5%** for the endothermic case, whereas the thermal profile increases for increasing magnitude of *E* by **1%** for the exothermic case and diminishes by **4%** for the endothermic case.
- The radiation parameter increases the thermal profile by **5.95%** in the ternary nanofluid, whereas for the nanofluid it increases by **4.85%**, which is a significant contribution to the enhanced thermal efficiency.
- The increase in the Schmidt parameter diminishes the concentration profile.

Aforementioned study discloses a complicated relation between various factors and their contribution towards the heat and mass transfer properties of the ternary hybrid nanofluid in comparison with the nanofluid. The current work is limited to the investigation of the effect of magnetised rotating ternary hybrid nanofluid with chemical reaction on the permeable stretching sheet. This work can be extended by considering different geometries, various nanofluid models, different combinations of nanoparticles, various physical conditions and boundary conditions.

## Data Availability

No data was used for the research described in the article.

## List of symbols

$$x,y,z$$: Coordinates $$(\text{m})$$
$a$: Stretching rate factor $({\text{s}}^{-1})$
$b$: Stagnation ratio factor $\left({\text{s}}^{-1}\right)$
$${u}_{w}$$: Stretching velocity $$\left({\text{ms}}^{-1}\right)$$
${u}_{e}$: Far-field velocity $\left({\text{ms}}^{-1}\right)$
$${T}_{w}$$: Surface temperature $$\left(\text{K}\right)$$
$${C}_{w}$$: Surface concentration
*C*: Concentration
*T*: Temperature (K)
$${T}_{\infty }$$: Ambient temperature $$\left(\text{K}\right)$$
$${C}_{\infty }$$: Ambient concentration
$$K$$: Boltzmann constant $$\left({\text{kgm}}^{2}{\text{s}}^{-2}{\text{K}}^{-1}\right)$$
${K}_{p}$: Porous medium permeability (${\text{m}}^{2}$)
$${E}_{A}$$: Activation energy $$({\text{kgm}}^{2}{\text{s}}^{-2})$$
$${Kr}^{2}$$: Chemical reaction ($${\text{s}}^{-1}$$)
$${C}_{p}$$: Specific heat $$({\text{Jkg}}^{-1}{\text{K}}^{-1})$$
$${C}_{b}$$: Drag force (m)
$${D}_{B}$$: Diffusivity ($${\text{m}}^{2}{\text{s}}^{-1}$$)
$${f}{\prime}$$: Dimensionless velocity
*Pr*: Prandtl number
*Rp*: Rotation parameter
*M*: Magnetic parameter
*Fr*: Forchheimer number
*Rd*: Radiation parameter
*E*: Activation energy parameter
*Rr*: Reaction rate parameter
*Sc*: Schmidt number
*n*: Fitted rate constant
*S*: Stretching parameter
${Re}_{x}$: Local Reynolds number
${C}_{fx}$: Skin friction in x-direction
${C}_{fy}$: Skin friction in y-direction
$${Nu}_{x}$$: Nusselt number
$${Sh}_{x}$$: Sherwood number
${k}^{*}$: Mean absorption coefficient

## Greek letters

$$\alpha $$: Thermal diffusivity $$({\text{m}}^{2}{\text{s}}^{-1})$$
$$\vartheta $$: Kinematic viscosity $$({\text{m}}^{2}{\text{s}}^{-1})$$
$$\theta $$: Dimensionless temperature
$\varepsilon$: Endothermic/exothermic reaction factor ${(\text{Jkg}}^{-1})$
$$\rho $$: Density $$\left({\text{kgm}}^{3}\right)$$
$$\Omega $$: Angular velocity ($${\text{s}}^{-1}$$)
$$\chi $$: Dimensionless concentration
$$\eta $$: Dimensionless variable
${\varpi }_{1}, {\varpi }_{2}, {\varpi }_{3}$: Solid volume fractions of nanoparticles
$$\sigma $$: Electrical conductivity (S $${\text{m}}^{-1}$$)
$$\mu $$: Viscosity (Pas)
${\sigma }^{*}$: Stefan-Boltzmann constant
$$\lambda $$: Porosity parameter
${\lambda }^{*}$: Endothermic/exothermic parameter
$\delta$: Temperature ratio parameter

## Subscripts

*F*: Fluid
*NF*: Nanofluid
*HF*: Hybrid nanofluid
*THNF*: Ternary hybrid nanofluid
